# Supplementary material for: Chromothripsis during telomere crisis is independent of NHEJ, and consistent with a replicative origin
Source: Genome Res. 2019 May;29(5):737–49. doi: 10.1101/gr.240705.118 (PMC6499312; doi:10.1101/gr.240705.118)
Supplement: Supplemental Material [file supp_gr.240705.118_Supplemental_file_1.zip › contigs/annotated_contigs/DB55/contig.2.DB55_length_265_mean_cov_6.62641509434.docx]

**DB55_length_265_mean_cov_6.62641509434**

```
TCTGTTGTCCAATCCTTCTTTAGTTTCTATACAGCACACATTCCTGATTGAAACCTTGCTCACTTATTTGGGGACGATATTGGGTTGAG
 >chr11:94765292-94765445 - E=6e-81
TGAATGAGTTATCAGTGTGAAAGCTACACACAGCTTTGAAAAGGCAGTGGAGCCCGGGGAG|AGA|TGCCTGAGAGTTCTGGGGAGTAA
                                                              >chr11:94758559-94758674
TTTCTTTTCTCCCCTTTGTTTTTCAAAATACTTTGGTTTATCTGTTAGATAGTAATTCACACTTGGACTTTTGAACAAGTGAGATCTTT- E=6e-52
```
